# Chloroprene and Butadiene Rubber (CR/BR) Blends Cross-Linked with Metal Oxides: INFLUENCE of Vulcanization Temperature on Their Rheological, Mechanical, and Thermal Properties

**DOI:** 10.3390/molecules30132780

**Published:** 2025-06-27

**Authors:** Aleksandra Smejda-Krzewicka, Konrad Mrozowski

**Affiliations:** Institute of Polymer and Dye Technology, Faculty of Chemistry, Lodz University of Technology, Stefanowskiego Street 16, 90-537 Lodz, Poland

**Keywords:** chloroprene rubber, butadiene rubber, elastomeric blends, cross-linking, interelastomeric bonds, vulcanization temperature, iron(III) oxide, copper(II) oxide

## Abstract

This paper aimed to evaluate the effect of cross-linking temperature on the rheological, mechanical, and thermal properties of CR/BR compositions cross-linked with zinc oxide, iron(III) oxide, or copper(II) oxide. Properties of CR/BR compounds were studied at four temperatures: 140, 160, 180, and 200 °C. The lowest activation energy of vulcanization was shown by blends cross-linked with ZnO, and the highest activation energy of vulcanization was shown by samples with Fe_2_O_3_. Blends cured with ZnO or Fe_2_O_3_ showed higher cross-linking activity than CuO. Higher temperatures enhanced the degree of cross-linking in the CR/BR composite cured with ZnO or CuO but slightly reduced it for the CR/BR/Fe_2_O_3_ vulcanizates. The highest tensile strength was observed for the CR/BR/Fe_2_O_3_ product. However, compositions cured with ZnO exhibited the best aging resistance. The CR/BR compounds cured with ZnO at high temperatures had the highest tear strength (16.8 N/mm), while samples containing CuO as a curing agent showed declining tear strength with temperature. DSC confirmed a single glass transition (~36 °C), indicating good elastomers dispersion. Infrared and SEM analyses confirmed effective cross-linking and blend compatibility.

## 1. Introduction

Elastomeric blends are important technologically and commercially because they enable the achievement of properties (mechanical, dynamic, thermal) of new materials that are not attainable with a single elastomer [1]. Present knowledge largely focuses on the selection of different types of elastomers and the creation of blends from them through both physical and chemical processes [2,3]. Compositions of elastomers can lead to changes in properties due to internal differences in formulation or differences in the reinforcement and method of vulcanization [1,4,5]. A common problem in creating blends is their limited miscibility, resulting in a microscopically heterogeneous phase structure of the elastomers [6,7]. Problems with the dispersibility of blends occur when dealing with elastomers that are immiscible with each other. Immiscible blends exhibit more complex changes due to the microscopically heterogeneous phase structure of the two-component elastomers. The two separate phases typically differ in the arrangement of the fillers and plasticizers in each other, as well as the cross-linking system [8,9]. This variation affects the cross-linking process and the engineering properties of the elastomers (i.e., tensile strength, hysteresis, tear resistance). Therefore, the amount of these additives in any phase can be modulated by changes in the viscosity and chemical identity of the elastomer, chemical composition, filler surface area, the chemical nature of the plasticizer, and the order in which the components are added and the mixing procedure used. In addition, immiscible blends require excellent phase dispersion and interfacial adhesion [8,10]. This is due to the need to ensure mechanical integrity in vulcanized elastomers.

In recent years, researchers have also been interested in blends of butadiene rubber (BR) with chloroprene rubber (CR) to produce high-performance elastomeric blends characterized by both good mechanical properties and resistance to low temperatures. Chloroprene rubber is characterized by unique mechanical properties, good adhesion to metals and textiles, susceptibility to thermal cross-linking, and resistance to certain technical media [11]. The presence of chlorine atoms in the main chain results in increased flame resistance of the elastomer and resistance to aging [12,13]. However, CR has several disadvantages, for example, poor ozone and UV resistance, which can cause cracking and surface degradation during prolonged exposure in outdoor applications. Poor flexibility, especially at low temperatures, and susceptibility to polar solvents are also disadvantages of chloroprene rubber [11]. These disadvantages can be overcome precisely by using blends with butadiene rubber [14,15]. Due to its excellent abrasion resistance, good wear resistance, and low rolling resistance, butadiene rubber (BR) is widely used in industry [16]. BR vulcanizates, due to their regular molecular structure and flexible chain, are characterized by low temperature and abrasion resistance, very good elasticity over a wide temperature range, and very good electrical insulating properties. Therefore, butadiene rubber is used to form blends with chloroprene rubber to improve its properties [17,18,19].

The biggest engineering challenge is the process of mixing BR with CR. Due to their chemical compositions, butadiene rubber (non-polar) and chloroprene rubber (polar) do not mix as easily and are difficult to disperse together to form a homogeneous matrix [17]. For this reason, there have been several publications in which researchers have sought a solution. It is worth mentioning that Mingyi et al. [20] investigated blends of butadiene rubber, chloroprene rubber, and styrene-butadiene-styrene copolymer (BR/CR/SBS). The results showed that the addition of a small amount of SBS copolymer to thermodynamically immiscible BR/CR blends significantly improved the homogeneity of the system and increased the cross-linking density of the vulcanizates and the tensile strength. Meanwhile, Menon and Visconte [21] studied the miscibility of CR/BR blends in the presence of phosphorylated cardanol prepolymer (PCP). They found that CR/BR blends in the presence of PCP had good compatibility and higher tensile strength and tear strength of the vulcanizates. The paper of Zheng et al. [22] presents low-temperature-resistant and electrically insulating CR/BR blends. The results show that the presence of BR significantly improves low-temperature-resistance and electrical insulation properties.

The method of cross-linking such compositions is important for the functional properties of CR/BR vulcanizates. A review of the literature found several publications describing the potential cross-linking of CR/BR blends and their properties. Słubik et al. [23] have shown that iron(III) oxide nanoparticles (nano-Fe_2_O_3_) can be used as a cross-linking agent for CR/BR compounds, and it enables controlled reactions between these elastomers. This vulcanization led to the production of products characterized by good mechanical properties (tensile strength, tear resistance) and very high fire resistance. CR/BR blends can also be cross-linked with iron(III) oxide (Fe_2_O_3_), iron(II,III) oxide (Fe_3_O_4_), silver(I) oxide (Ag_2_O), or zinc oxide (ZnO) [18]. The results showed that all vulcanizates, regardless of the oxide used, were characterized by a high degree of cross-linking and satisfactory mechanical properties due to the formation of intermolecular bonds between CR and BR. The most important advantages of the obtained rubber products are very high flame resistance, simple technology, and low cost of obtaining flame-retardant materials. CR/BR compositions can also be cured with zinc nano-oxide (nZnO), in the presence of silica fillers [24]. The results showed that these vulcanizates exhibited good mechanical properties, good damping properties, and non-flammability. In this study, it was not possible to achieve a homogeneous structure of the materials obtained, but the addition of fillers allowed satisfactory properties. Among these publications, different types of oxides and fillers and their effects on the properties of the produced materials have been studied. However, the effect of vulcanization temperature on the cross-linking process and properties of the CR/BR blends has not been explored.

This research focuses on evaluating the effect of vulcanization temperature on the cross-linking of elastomeric blends and the properties of their products. The objects of the study were blends of chloroprene rubber and butadiene rubber cross-linked with different curing agents such as zinc oxide (ZnO) [25], iron(III) oxide (Fe_2_O_3_) [26], or copper(II) oxide (CuO) [27]. Cross-linking mechanisms with metal oxides at selected temperatures have been precisely described in our previous papers, but the influence of vulcanization temperature on the cross-linking of the mentioned compositions and the properties of vulcanizates have not yet been investigated. Therefore, this study fits into the need to correlate curing conditions with the mechanical, thermal, and chemical properties of CR/BR blends. This research is important in the context of industrial-scale development of CR/BR blends. It gives information on potential mechanisms and the effects of metal oxide type and temperature on the properties of vulcanizates.

## 2. Results and Their Discussion

### 2.1. Cross-Linking Kinetics Analysis

**Figure 1:**
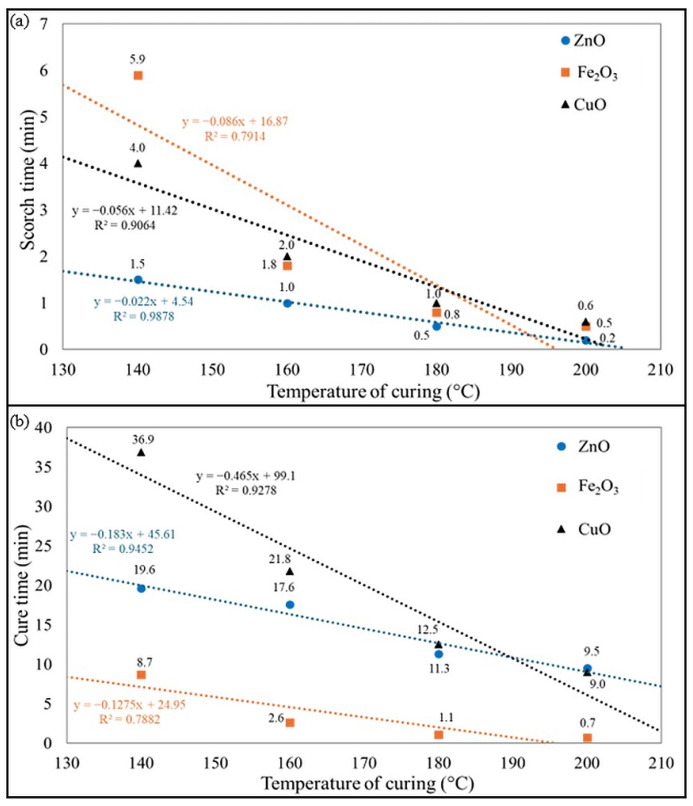
Effect of curing agent and temperature on: (**a**) scorch and (**b**) cure time of CR/BR blends.

Assessing vulcanization kinetics provides important insights into the behavior of elastomers during cross-linking. Rheometers measure torque growth over time, indicating the progress of the cross-linking reaction. Parameters such as scorch time (t_02_), curing time (t_90_), and torque increment after selected time (ΔT) are analyzed to evaluate cure rate and cross-linking density. By testing these kinetics at different temperatures and with different curing systems, researchers can optimize processing conditions to achieve the desired mechanical and thermal properties of elastomeric compounds. The results of the vulcanization kinetics of the tested CR/BR blends are summarized in Table 1. Analysis of these parameters allowed preliminary characterization of the resulting vulcanizates. The t_90_ parameter represents the optimal curing time, indicating the duration required for the rubber compound to reach its maximum torque. A higher t_90_ value implies a longer vulcanization process, consequently extending the overall processing duration. Moreover, scorch time (t_02_) signifies the onset of vulcanization, marking the moment at which the curing process begins. This value is crucial for ensuring the safe processing of rubber before the vulcanization reaction becomes significant. Both parameters depend on temperature and curing system; therefore, Figure 1 shows the effect of temperature and the cross-linking agent used on the scorch time and cure time values.

The use of zinc oxide in CR/BR blends allowed the achievement of great values of the torque increment in a short time. As the temperature increased, the scorch and vulcanization times decreased. It is worth noting that the CR/BR/ZnO blends were characterized by similar ΔT_10_ values. Moreover, it is worth pointing out that the higher the cross-linking temperature, the greater the torque increment. However, this growth is not as spectacular in the case of blends cross-linked with iron(III) oxide. For these blends, the higher temperature resulted in a shorter vulcanization time. It should be noted that blends cured at 140 °C achieved optimal vulcametric parameters and recorded the highest torque increments, which were responsible for a high degree of cross-linking. These compositions should be considered in the context of more sustainable vulcanization because the typical vulcanization temperature for rubber is 160 °C, but for this sample, the best properties were obtained when curing at 140 °C. This is an advantageous aspect in the context of less emissive elastomeric blends since a lower curing temperature means that less energy is required for the process. In the case of the CR/BR/CuO blends, the vulcanization time decreased with increasing temperature (from 36.9 min at 140 °C to 9 min at 200 °C). A similar trend was observed for the scorch time. Torque increments after 20 min also obtained greater values for cross-linking at higher temperatures, so using CuO as a curing agent was more effective at higher temperatures.

**Figure 2:**
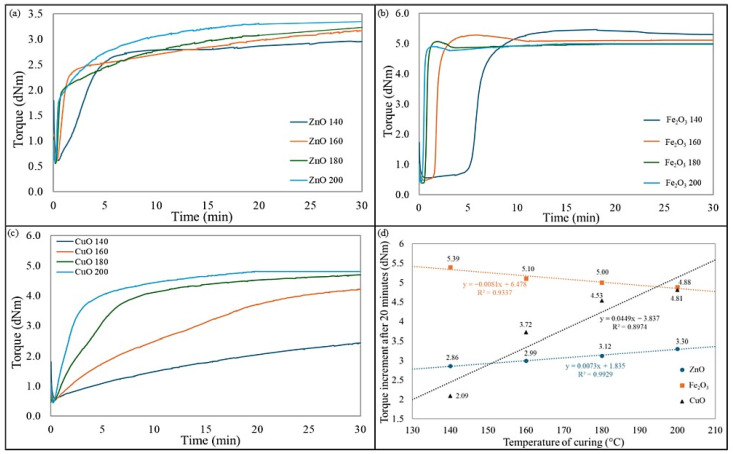
Curing kinetics curves for (**a**) CR/BR/ZnO, (**b**) CR/BR/Fe_2_O_3_, and (**c**) CR/BR/CuO; and (**d**) influence of curing system and temperature on torque increment after 20 min of CR/BR blends.

For the CR/BR/ZnO blends, a significant change in vulcanization time is noted between 160 °C and 180 °C. In the case of the CR/BR/Fe_2_O_3_ composites, a substantial difference occurred when cross-linking at temperatures of 160 °C and above, with an almost threefold reduction. Figure 2 illustrates kinetics curves and changes in torque increment after 30 min under different curing temperatures and systems. Samples cross-linked with iron(III) oxide exhibited stable values, with the highest torque increment at 140 °C, while for other temperatures, a slight decrease was observed. In contrast, for samples cross-linked with ZnO or CuO, the opposite trend is noted (i.e., the higher the temperature, the greater the value of torque increment). For the CR/BR/ZnO blends, ΔT_20_ increased from 2.86 dNm to 3.30 dNm. However, for samples cross-linked with CuO, a more than twofold increase in ΔT_20_ values is observed, rising from 2.09 dNm to 4.81 dNm (Figure 2d). Analyzing the behavior of the curves in Figure 2a–c, it can be concluded that blends cross-linked with Fe_2_O_3_ do not need high temperatures to vulcanize optimally. The vulcametric curves for CR/BR/ZnO and CR/BR/CuO compositions vulcanized at 140, 160, 180, and 200 °C showed marching characteristics, while the CR/BR/Fe_2_O_3_ composition tended to reverse for all temperatures. It is also worth noting that the minimum torque (T_min_), indicating the viscosity of tested CR/BR blends, did not depend significantly on the type of metal oxide used as a cross-linking agent. The T_min_ value for all compositions cured at 140 °C was 0.6 dNm. This parameter confirms that the viscosity of the CR/BR composites was comparable regardless of the curing substance used. The T_min_ values decreased slightly if the CR/BR blends were vulcanized at higher temperatures.

Table 2 summarizes the values of the activation energies of scorch or vulcanization calculated from the Arrhenius equation. The calculated values show that the cross-linking of blends was an exothermic reaction. The values of the activation energies of scorch and vulcanization were the lowest (E_scr_.−54.28 kJ/mol, E_vulc_.−21.23 kJ/mol) for the composition containing zinc oxide, which may be related to the smaller size of its particles and the lower energy requirement for the vulcanization process. For the composition cured with iron(III) oxide, the activation energies of scorch and vulcanization were close to each other, which means that once the energy barrier for scorch is exceeded, the blend begins to cross-link quickly (Figure 2a–c), so this oxide was very active. In the case of the CR/BR/CuO blend, the value of the activation energy of the scorch is the highest, which means that a higher energy barrier needs to be crossed to start cross-linking. This is also confirmed by vulcametric curves.

### 2.2. Structural and Morphological Characterization of CR/BR Composites

**Figure 3:**
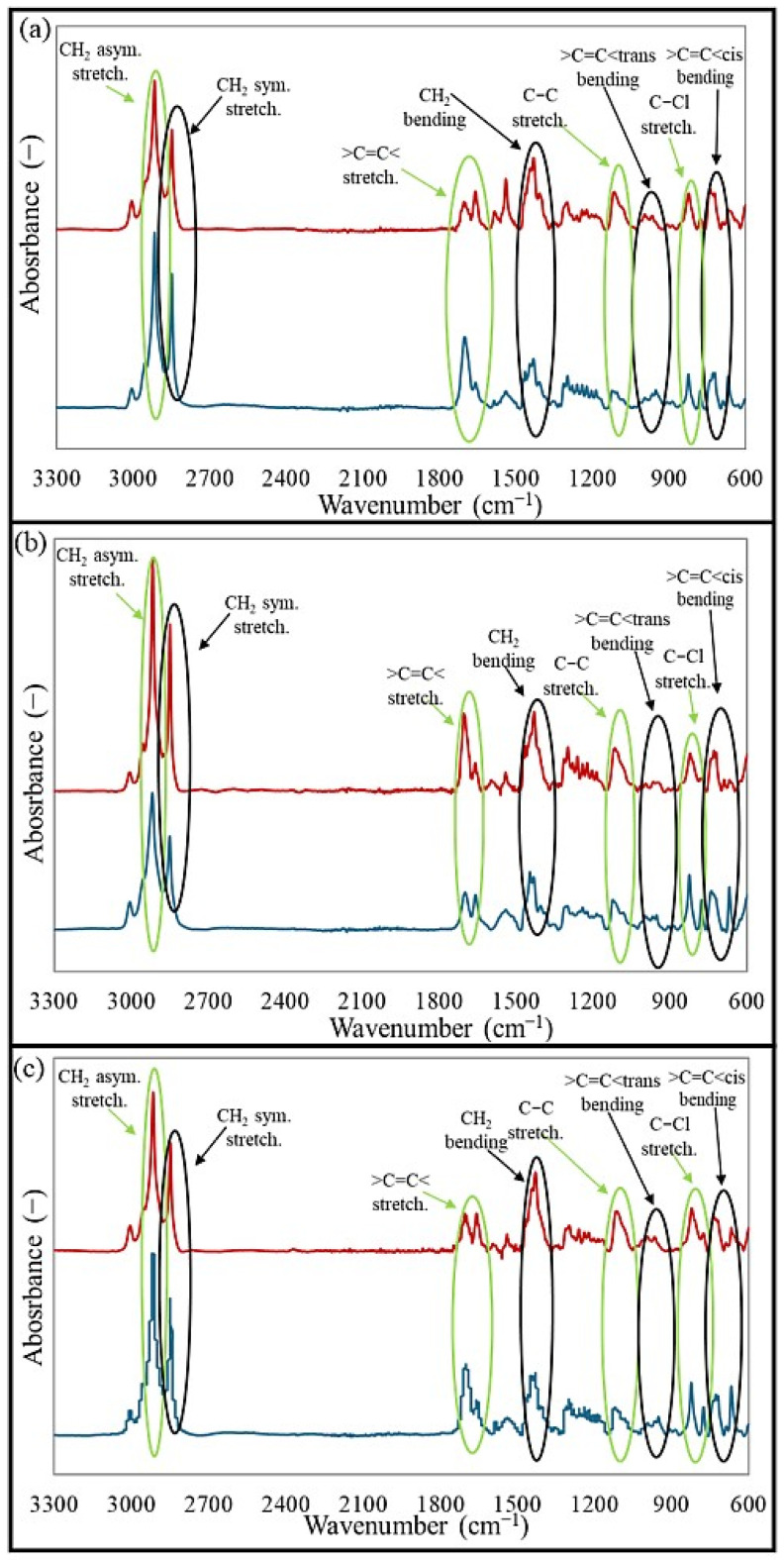
FT-IR spectra: (**a**) CR/BR/ZnO, (**b**) CR/BR/Fe_2_O_3_, (**c**) CR/BR/CuO cross-linked at 200 °C. Red line—blend, blue line—vulcanizate.

Fourier transform infrared spectroscopy (FT-IR) is widely used in polymer analysis because it provides valuable information on the chemical structure and composition of polymers. The method detects specific vibrations of molecular bonds in a polymer, enabling the identification of functional groups. In this study, the analysis of FT-IR spectra aims to determine whether any interelastomeric bonds were formed after vulcanization and to assess changes in the intensity of absorption signals. Fourier transform infrared spectroscopy was performed to analyze the structures of the studied blends and vulcanizates obtained during the cross-linking at 200 °C. In the obtained spectra, illustrated in Figure 3, the intensity of the bands for both the blends and vulcanizates in the wavenumber range of 2945–2840 cm^−1^ corresponded to the asymmetric and symmetric stretching of -CH_2_- groups [28,29]. The intensities of vibrations in the region of 1660–1656 cm^−1^ corresponded to >C=C< stretching [30], which decreased for the CR/BR/ZnO and CR/BR/CuO samples cross-linked at 200 °C. This decrease may be related to the breaking of double bonds in macromolecular chains, followed by the formation of interelastomeric bonds. In the area of 1430–1420 cm^−1^, the increase in band intensity, caused by changes in the structure of the vulcanizates, is associated with -CH_2_- deformations [31,32]. An increase in absorption for the vulcanizates was observed in the range of 1123–1121 cm^−1^, attributed to the stretching of C-C bonds in the main macromolecular chains [33], which resulted from structural reorganization during the cross-linking with oxides at elevated temperatures. In the region of 996–950 cm^−1^, bands corresponding to C-H deformation vibrations in the >CH=CH<_trans_ configuration were observed [34,35], while in the absorption range of 689–668 cm^−1^, peaks characteristic of C-H deformation vibrations in the >CH=CH<_cis_ configuration were detected [36,37]. The increase in trans units in the studied vulcanizates is probably related to the formation of intermolecular bonds between butadiene rubber and chloroprene rubber. The reduction in absorption band intensity at 825–820 cm^−1^ for vulcanizates (C-Cl stretching vibrations) is associated with elastomer cross-linking. FT-IR spectral analysis suggests that the type of oxide used for cross-linking does not significantly affect band intensity, indicating the formation of a comparable network structure [32].

**Figure 4:**
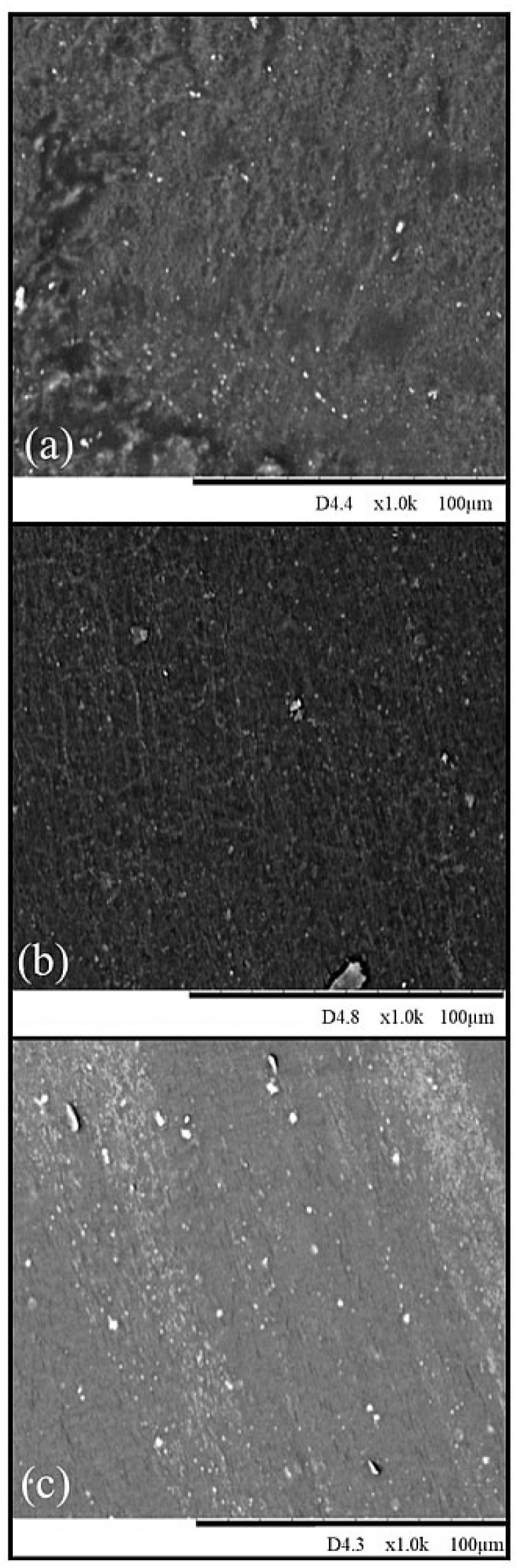
SEM images of surface: (**a**) CR/BR/ZnO, (**b**) CR/BR/Fe_2_O_3_, and (**c**) CR/BR/CuO cross-linked at 200 °C.

The study of the structure and morphology of materials is an important aspect of materials science. It involves understanding the composition and chemistry related with processing, micro- or nano-structure, and properties. Analysis of SEM images enabled checking the compatibility of non-polar butadiene rubber with polar chloroprene rubber and evaluating the effect of the cross-linking agent (ZnO, Fe_2_O_3_, and CuO) on the surface morphology of the vulcanizates. The SEM images of composites cross-linked with zinc oxide, iron(III) oxide, and copper(II) oxide at 200 °C are shown in Figure 4 (at 1000× magnification). It is noted that the surfaces of the samples were homogeneous and there was no visible separation of the rubber phases, which indicates the activity of the cross-linking substances interacting between the two elastomers in the tested CR/BR blends. The surface of the sample cured with zinc oxide was rough and contained oxide clusters (dark fragments) (Figure 4a). The composition containing iron(III) oxide had cracks on its surface (Figure 4b). In contrast, the composition cross-linked with copper(II) oxide (Figure 4c) had small and regularly spaced particles in the elastomeric matrix on its surface.

### 2.3. Analysis of the Degree of Cross-Linking Determined by the Equilibrium Swelling

Swelling properties are crucial for elastomeric products as they determine the material’s resistance to solvents, oils, and chemicals, directly affecting performance and durability. Excessive swelling can lead to softening, loss of mechanical strength, and dimensional instability, necessitating the optimization of cross-link density and curing conditions. Swelling behavior analysis helps predict long-term stability, chemical resistance, and compatibility with different environments, which is particularly important for seals, gaskets, fuel hoses, and industrial applications.

The obtained results of equilibrium swelling are presented in Table 3. For the CR/BR/ZnO vulcanizate, the highest equilibrium volume swelling in toluene (Q_V_^T^ = 8.24 mL/mL) was observed for the CR/BR/ZnO-160 sample, followed by a slightly lower value (Q_V_^T^ = 8.06 mL/mL) for the CR/BR/ZnO-140 vulcanizates. As the curing temperature increased (180 and 200 °C), the Q_V_^T^ values decreased, indicating a higher cross-link density and a lower amount of unbound rubber, which swells upon contact with the solvent. For the CR/BR/Fe_2_O_3_ composites, the lowest equilibrium swelling value in toluene (Q_V_^T^ = 5.63 mL/mL) was obtained at the lowest curing temperature, while increasing temperature led to higher Q_V_^T^ values. The greatest equilibrium volume swelling in toluene (Q_V_^T^ = 13.61 mL/mL) was found for the CR/BR/CuO-140 vulcanizate. For the composite cured with CuO at 160 °C, the equilibrium swelling in toluene was significantly lower (Q_V_^T^ = 7.35 mL/mL). As the curing temperature of CR/BR/CuO blends increased, the equilibrium volume swelling in toluene decreased, with the lowest value (Q_V_^T^ = 5.14 mL/mL) observed at 200 °C. For swelling in n-hexane, a similar trend was observed for CR/BR/ZnO vulcanizates, where the highest volume swelling (Q_V_^H^ = 1.28 mL/mL) was obtained for the sample cured at 160 °C, and with higher temperatures (180 and 200 °C), the Q_V_^H^ values decreased. The equilibrium swelling values in n-hexane for all CR/BR/Fe_2_O_3_ vulcanizates were comparable (~1 mL/mL). However, for the CR/BR/CuO products, Q_V_^H^ decreased with increasing temperature, with the lowest value (Q_V_^H^ = 0.77 mL/mL) recorded for the composite cured at 200 °C.

**Figure 5:**
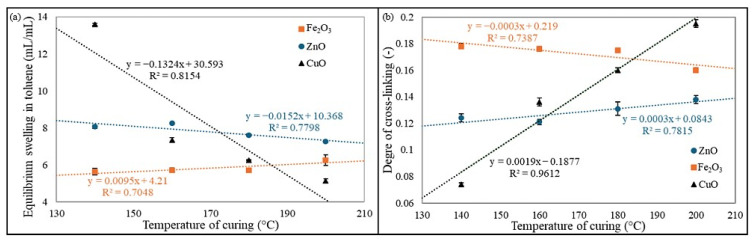
Impact of curing system and temperature on (**a**) equilibrium volumetric swelling in toluene and (**b**) degree of cross-linking of CR/BR vulcanizates.

The leached fraction eluted in toluene was higher than in n-hexane, indicating that the tested elastomeric composites were more sensitive to toluene. The highest values (−Q_W_^T^ = 0.31 mg/mg) were recorded for the CR/BR/CuO-140 vulcanizate, while the lowest (−Q_W_^T^ = 0.13 mg/mg) was for the CR/BR/CuO-200 sample. For blends cross-linked with ZnO or CuO, the values decreased with increasing temperature, while for the CR/BR/Fe_2_O_3_ products, no important changes were observed. Moreover, the leached fraction eluted in n-hexane, ranging from 0.05 to 0.08 mg/mg for all tested samples. The rubber volume fraction in the swollen sample immersed in toluene increased with curing temperature for CR/BR/ZnO and CR/BR/CuO samples but decreased for CR/BR/Fe_2_O_3_ vulcanizates. A higher rubber volume fraction indicates a greater degree of cross-linking. The lowest V_R_^T^ value in toluene (0.0685) was observed for the sample cured with CuO at 140 °C, while the highest V_R_^T^ value (0.163) was recorded for the CR/BR/CuO-200. The highest degree of cross-linking for samples tested in toluene (α_c_^T^ = 0.195) was obtained for the CR/BR/CuO-200 vulcanizate, while the lowest α_c_^T^ value (0.0735) was calculated for the CR/BR/CuO-140 composite. For blends cross-linked with CuO and ZnO, higher curing temperatures resulted in higher α_c_ values, while an opposite trend was observed for CR/BR/Fe_2_O_3_ samples. Figure 5 shows the relationship between cross-linking temperature and curing agent and such values as Q_v_^T^ and α_c_.

**Figure 6:**
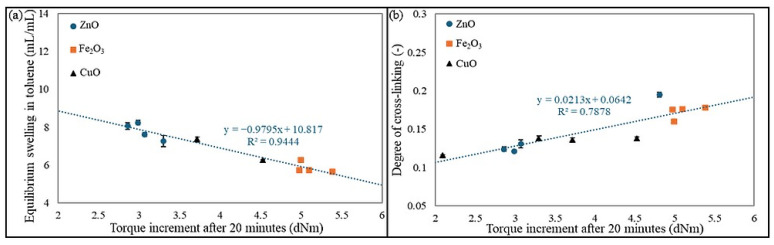
Relationship of torque increment to (**a**) equilibrium volumetric swelling in toluene and (**b**) degree of cross-linking.

The equilibrium swelling results indicate that the CR/BR blend cross-linked with copper(II) oxide at 200 °C exhibited the highest resistance to solvents, confirming a well-developed cross-linking network in this vulcanizate. Additionally, as the curing temperature of the CR/BR/CuO blend increased, the Q_V_^T^ and Q_V_^H^ values decreased, indicating a higher degree of cross-linking and the formation of a more complex network structure. For samples cross-linked with iron(III) oxide, the opposite trend was observed. However, these differences were less pronounced than those observed for CR/BR/CuO products. Figure 6 shows the correlation of equilibrium swelling and degree of cross-linking with the torque increment after 20 min for all tested samples. Figure 6a indicates that the higher the ΔT_20_ value, the lower the Q_v_^T^ parameter, which is related to the higher number of cross-links in the materials and affects the lower possibility of solvent penetration into the material [38,39]. Meanwhile, Figure 6b shows that the larger the ΔT_20_ value, the greater the degree of cross-linking, resulting from the increasing number of cross-links [40].

### 2.4. Analysis of the Degree of Cross-Linking Determined by the Elasticity Constants

The next method used in the study to determine the networks formed during the cross-linking was the determination of the Mooney–Rivlin constants. The value of the first elasticity constant (2C_1_) is directly proportional to the degree of cross-linking (the higher its value, the greater the degree of cross-linking of the vulcanizate), while the second elasticity constant (2C_2_) is a measure of the deviation of the formed network from an ideal network [41]. The obtained test results are presented in Table 4.

After analyzing the study, it should be noted that for compositions cross-linked with ZnO or CuO, the 2C_1_ values increase with increasing vulcanization temperature, indicating a higher degree of cross-linking in these samples. For the CR/BR/Fe_2_O_3_ vulcanizate, no significant trend was observed. Moreover, for all temperatures, the CR/BR/Fe_2_O_3_ vulcanizates exhibited similar 2C_1_ values (3.21–4.03 kG/cm^2^). Comparable to previous studies, the highest degree of cross-linking was obtained for the CR/BR/CuO sample cross-linked at 200 °C, where the first elasticity constant was 4.25 kG/cm^2^. For the CR/BR/CuO sample cross-linked at 160 °C, a similar value of the first elasticity constant (3.61 kG/cm^2^) was observed as for the CR/BR/ZnO-180 sample (3.67 kG/cm^2^). However, for the sample containing copper(II) oxide, the value of the second elasticity constant was significantly higher (4.54 kG/cm^2^), which may suggest that structures are formed in this vulcanizate that hinder the creation of an ideal network despite the high degree of cross-linking. Additionally, these structures contribute to the strain-hardening effect, making the material more resistant to stretching under large deformations [42].

**Figure 7:**
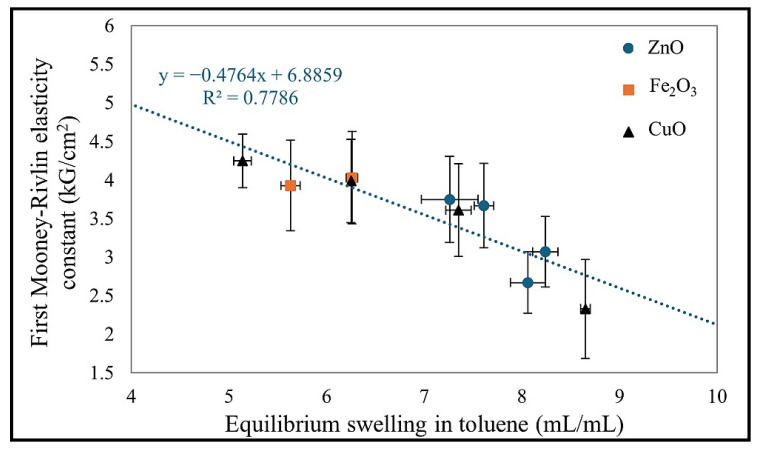
Influence of equilibrium volumetric swelling in toluene with the first Mooney–Rivlin elasticity constant.

Furthermore, the 2C_2_ parameter in the Mooney–Rivlin model suggests that the material behaves similarly to a neo-Hookean rubber, exhibiting a simpler elastic response with minimal strain stiffening [43]. The results for the CR/BR/Fe_2_O_3_-200 demonstrated greater resistance to high deformations (2C_2_ = 2.63 kG/cm^2^), while the remaining samples exhibited lower network stiffening and behaved more like neo-Hookean rubbers [42,43]. This means that at low deformations, the stress-strain relationship remains nearly linear. This behavior aligns with the formation of a more uniform cross-linked network, resulting in a predictable, linear stress-strain response [44]. Figure 7 represents the correlation of the first elasticity constant with the value of the equilibrium volume swelling in toluene for all the samples tested. The value of Q_v_^T^ is inversely proportional to the value of 2C_1_.

### 2.5. Mechanical Properties of Vulcanizates Before and After Thermo-Oxidative Aging

The tensile strength properties before and after thermo-oxidative aging of the vulcanizates are summarized in Table 5. The mechanical properties of rubber materials are directly related to cross-linking parameters. The conducted studies showed that the type of cross-linking agent used and the vulcanization temperature affect the tensile strength properties. The mechanical properties of elastomeric products before and after aging are crucial for assessing their durability and long-term performance. Before aging, properties such as tensile strength, elongation at break, and hardness indicate the initial quality and suitability of the material for specific applications. After aging, changes in these properties reflect the material’s resistance to thermal, oxidative, and environmental degradation. This evaluation helps predict the lifespan, mechanical stability, and risk of failure in applications such as automotive seals, tires, and industrial components.

**Figure 8:**
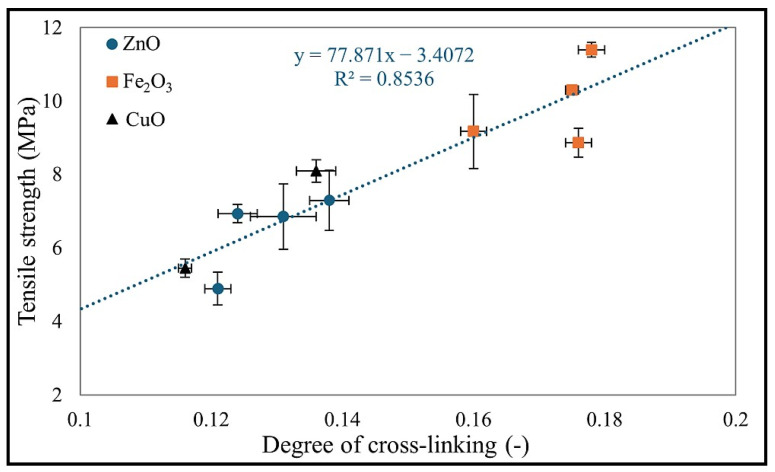
Relationship between degree of cross-linking and tensile strength of CR/BR vulcanizates.

The results of mechanical tests showed that the best mechanical properties (TS_b_ = 11.4 MPa) were achieved by the CR/BR/Fe_2_O_3_-140. Additionally, it is worth noting that samples cross-linked with Fe_2_O_3_ generally exhibited the best mechanical parameters, regardless of the curing temperature. Samples cured with ZnO achieved the highest tensile strength (7.29 MPa) at 200 °C. Vulcanizates containing CuO had the highest TS_b_ value (9.17 MPa) at 180 °C. Surprisingly low strength (TS_b_ = 2.59 MPa) was observed for the composition cross-linked with CuO at 200 °C. It is important to mention that cross-linking temperatures affect the mechanical properties of the studied systems differently, making it difficult to identify a clear trend. However, optimal curing temperatures can be indicated for specific elastomeric blends: 200 °C for CR/BR/ZnO, 140 °C for CR/BR/Fe_2_O_3_, and 180 °C for CR/BR/CuO. When analyzing the elongation at break (E_b_), all samples (except the CR/BR/CuO-200 compound) exhibited elongation above 800%, whereas the CR/BR/CuO-200 vulcanizates stretched only to 393%. Such a low value indicates over-cross-linking of the composition and a lack of balance between elasticity and flexibility. This is further supported by the S_E100_ value of 0.94 MPa, the highest among the tested samples, despite the lowest TS_b_ value. The CR/BR/CuO-200 vulcanizate had the highest degree of cross-linking (α_c_ = 0.195) among all samples and was characterized by increased stiffness while simultaneously reducing mechanical strength. Figure 8 shows the relationship between the degree of cross-linking and the tensile strength values of the CR/BR vulcanizates tested. It should be noted that, in general, tensile strength increased in direct proportion to the degree of cross-linking. This is because a higher number of cross-links creates a tighter polymer network, improving stress transfer between polymer chains. It also affects less chain slippage, making the material stronger under tension [45,46,47]. However, not all samples fulfilled this relationship. An example is the CR/BR/CuO-200 compound mentioned early on, which is over-cross-linked [48].

**Figure 9:**
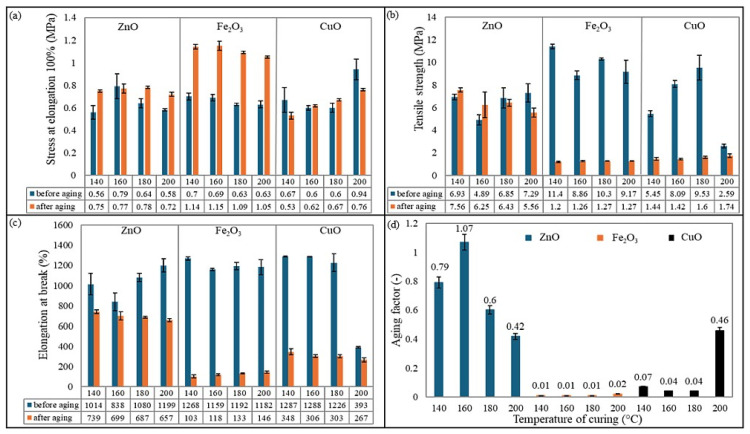
Comparison of mechanical properties before and after thermo-oxidative aging and aging resistance of CR/BR vulcanizates: (**a**) stress at elongation 100%, (**b**) tensile strength, (**c**) elongation at break, and (**d**) aging factor.

The mechanical strength analysis after thermo-oxidative aging revealed low resistance to aging factors in the samples cross-linked with CuO or Fe_2_O_3_. The mechanical properties of these vulcanizates significantly deteriorated after thermo-oxidative aging. For samples cross-linked with iron(III) oxide, the TS^′^_b_ value was approximately 1.2 MPa, with an aging coefficient of ~0.01. In the case of CR/BR/CuO samples, it was observed that the vulcanizate at 200 °C had an aging coefficient (~0.46) that deviated significantly from those obtained at other temperatures (~0.06). This discrepancy arises from the fact that, compared to the other samples, the tensile strength and elongation at break before thermo-oxidative aging were very low, possibly due to exceeding the cross-linking threshold and initiating sample degradation. As a result, a significant decline in these values after aging was not observed. Additionally, S^′^_E100_ values increased, indicating polymer chain stiffening and reduced deformation ability. However, this had a significantly negative effect on the tensile strength values after aging. It can therefore be concluded that these metal oxides, due to their variable valency, have a significant impact on the aging resistance of CR/BR vulcanizates. Transition metal oxides (Fe_2_O_3_, CuO) with variable oxidation states can act as redox catalysts in the presence of heat and oxygen, accelerating the decomposition of hydroperoxides into free radicals (RO•, HO•) [49]. These radicals cause chain scission, leading to a loss of mechanical properties, accelerated oxidative degradation, and rubber embrittlement [17,18,50,51]. Tensile strength after thermo-oxidative aging increased for CR/BR/ZnO blends cross-linked at 140 and 160 °C, possibly due to additional cross-linking of the samples. However, for samples cured with ZnO at 180 or 200 °C, tensile strength decreased, which corresponded to a decrease in the aging coefficient (0.60 and 0.42, respectively). The CR/BR/ZnO-160 vulcanizates were the most aging resistant, and their aging coefficient was approximately 1.0. This high aging resistance is attributed to the fact that zinc, unlike copper and iron, has a filled “d” subshell, making it more stable and preventing it from catalyzing aging processes. Conversely, since iron and copper atoms exist in multiple oxidation states, CR/BR blends cross-linked with these oxides exhibit poorer aging resistance compared to samples containing ZnO [19,24]. Figure 9 summarizes comparisons of the mechanical parameters before and after thermo-oxidative aging of the tested elastomer compositions (Figure 9a–c), as well as the aging coefficient values for all the samples tested.

**Figure 10:**
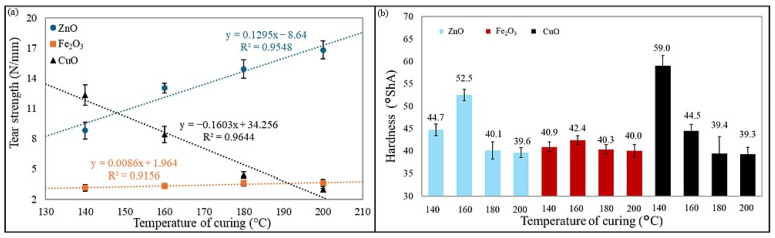
Effect of curing temperature and curing system on (**a**) tear strength and (**b**) hardness.

Hardness is another important mechanical parameter, as it directly affects wear resistance, load-bearing capacity, and elasticity. It determines how well an elastomer can withstand deformation under applied forces. Optimal hardness provides a balance between elasticity and durability, preventing excessive deformation or brittleness. Hardness is widely used for an indicative assessment of rubber and the degree of vulcanization, and it depends on the elastic properties of the material under test (Young’s modulus) [52,53]. The CR/BR/CuO-140 vulcanizate was characterized by the highest hardness (59 °Sh A), and the lowest hardness (39.3 °Sh A) was observed for the CR/BR/CuO-200 product. The highest hardness values for CR/BR/ZnO and CR/BR/Fe_2_O_3_ vulcanizates were obtained for samples cured at 160 °C (52.5 °Sh A and 42.4 °Sh A, respectively). Figure 10 presents the effect of the curing system and curing temperature on the tear strength of the samples, as well as a comparison of the hardness of the CR/BR vulcanizates tested.

Tear strength is a critical property of elastomeric compounds, reflecting their resistance to crack initiation and propagation under mechanical stress. This is particularly important for applications exposed to repeated bending, stretching, and impact. High tear strength enhances durability, reliability, and service life, preventing premature failure under demanding conditions. The tear strength of rubber materials is measured as the force required to tear a pre-cut sample and is expressed as the ratio of the maximum force causing sample failure to its thickness. Among the tested materials, the CR/BR/ZnO-200 vulcanizate exhibited the highest tear resistance (16.8 N/mm), whereas the CR/BR/CuO composite cured at 200 °C showed the lowest tear strength (2.95 N/mm). For the CR/BR/ZnO composition, tear strength increased with rising vulcanization temperature, while an inverse trend was observed for the CR/BR/CuO samples. The CR/BR/Fe_2_O_3_ vulcanizates demonstrated the lowest tear strength (2.6–3.6 N/mm).

### 2.6. Analysis of Thermal Properties

Thermal properties, as analyzed by differential scanning calorimetry (DSC) and thermogravimetric analysis (TGA), are crucial for understanding the stability and performance of elastomeric blends under various temperature conditions. DSC helps determine glass transition temperature (T_g_), melting behavior, and phase transitions that affect flexibility and processing. TGA provides insight into thermal degradation, weight loss, and thermal stability, essential for predicting material life and high temperature resistance. These analyses help optimize elastomer formulations for heat resistance, processing conditions, and long-term durability in demanding applications.

**Figure 11:**
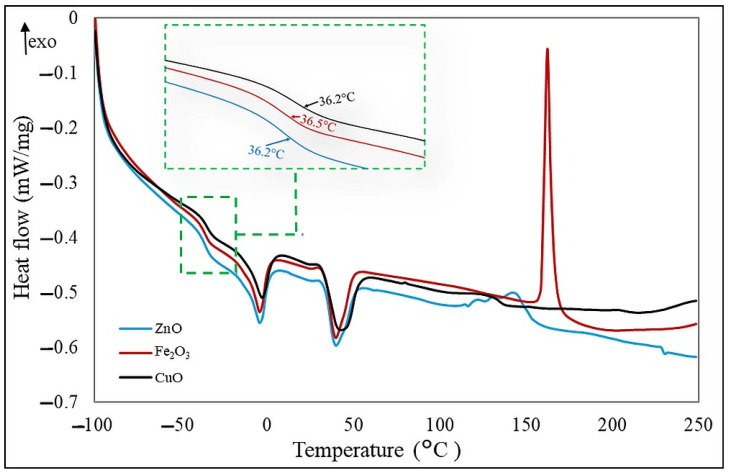
DSC curve of the CR/BR blends.

The glass transition temperature (T_g_) values obtained for the compositions tested are shown in Table 6. For all samples, the T_g_ values do not differ significantly. The type of cross-linking agent added to the CR/BR compositions caused changes in the cross-linking temperatures. The presence of zinc oxide or copper(II) oxide in the blends caused a shift in the exothermic peak, corresponding to cross-linking, toward a lower temperature than when iron(III) oxide was used. However, the type of oxide used had no noticeable effect on the change in glass transition temperature. Appearing exothermic peaks in the temperature ranges in Table 6 indicated cross-linking of the samples. Cross-linking temperatures show the largest values of the peaks, which were where the cross-linking process occurs most rapidly. The use of iron(III) oxide results in more efficient cross-linking of the elastomeric mix, as evidenced by the increasing amount of energy released during the process. The lowest enthalpy of cross-linking was determined for the sample cured with copper(II) oxide, which means that cross-linking in this case is the least effective. Figure 11 shows DSC curves for the three tested elastomeric blends differing in curing system.

**Figure 12:**
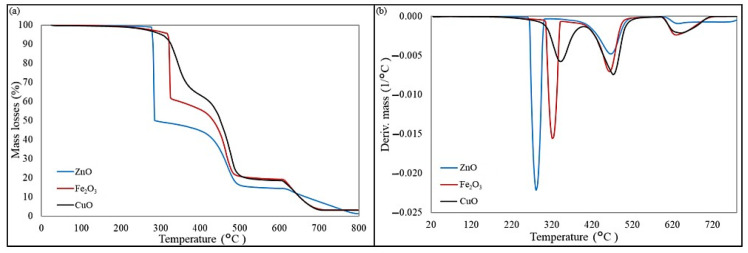
Effect of curing system on thermal properties of CR/BR blends: (**a**) TGA curves, and (**b**) DTG curves.

Figure 12a shows the TGA curve, which identifies the lost weight of the sample as a function of temperature and illustrates the thermal stability of the material. For the composition cross-linked with zinc oxide, the first stage of thermal decomposition began above 285 °C and was 49.8%. The use of iron(III) oxide as a cross-linking agent increased the decomposition temperature of the samples to 325 °C, and the weight loss was 37.9%. The elastomeric composition with the addition of copper(II) oxide is pyrolyzed at 345 °C. The first stage of decomposition for all samples ended at about 420 °C, and then the course of the thermogravimetric curves is almost identical. In Figure 12b, the mass loss during the first stage could be seen as the first peak and was mainly due to the elimination of HCl. In the second stage of decomposition taking place in the temperature range of 420–500 °C, a less rapid mass loss of the CR/BR/ZnO and CR/BR/Fe_2_O_3_ samples is noticeable than in the first stage, while the initial mass loss is greater for the CR/BR/CuO sample. At ~620 °C, there was a final initial weight loss of 18–20% for the samples. In summary, the CR/BR/ZnO product had the highest weight loss in the first stage (49.8%), while the CR/BR/CuO composite had the lowest weight loss (37.9%). The differences in the weight loss may be due to the thermal stability of their cross-linking agents. Therefore, the use of iron(III) oxide or copper(II) oxide improved thermal stability, but these compositions aged faster than zinc-oxide-cured blends after extended thermal exposition, confirming the mechanical properties after thermo-oxidative aging.

## 3. Materials and Methods

### 3.1. Materials

To produce elastomeric blends, butadiene rubber (BR; type: SYNTECA^®^44, containing 97% cis-1,4-butadiene mers with Mooney viscosity: ML 1 + 4 (100 °C): 39–49, obtained from Synthos S.A. (Oświęcim, Poland)) and chloroprene rubber (CR; type: BAYPREN^®^216, containing 40% bound chlorine, with Mooney viscosity: ML 1 + 4 (100 °C): 49 ± 5), received from LANXESS GmbH (Köln, Germany), were used. Zinc oxide (ZnO), with a density of 5.68 g/cm^3^ and a particle size of <100 nm; iron(III) oxide (Fe_2_O_3_), with a density of 5.25 g/cm^3^ and a particle size of <5 μm; and copper(II) oxide (CuO), with a density of 6.32 g/cm^3^ and a particle size of <10 μm, were used as the cross-linking system, all supplied by Sigma-Aldrich (St. Louis, MO, USA). Stearic acid, with a density of 0.85 g/cm^3^, delivered by Chempur (Piekary Śląskie, Poland), was used as a dispersing agent.

As solvents to study the degree of cross-linking, the following substances were used: toluene with a density of 0.87 g/cm^3^, delivered by POCh S.A. (Gliwice, Poland); n-hexane with a density of 0.66 g/cm^3^, sourced by POCh S.A. (Gliwice, Poland); and diethyl ether with a density of 0.71 g/cm^3^, obtained from Chempur (Piekary Śląskie, Poland).

### 3.2. Compounding and Vulcanization of CR/BR Blends

Each of the blends prepared included two rubbers, butadiene (BR), chloroprene (CR), and stearic acid. Various oxides were used to cross-link the CR/BR blends: zinc oxide (ZnO), iron(III) oxide (Fe_2_O_3_), or copper(II) oxide (CuO). The detailed composition of all blends is given in Table 7. The proportions of the components were chosen based on previous tests and research [9,18,19,23] to achieve the most optimal properties for the blends tested.

**Figure 13:**
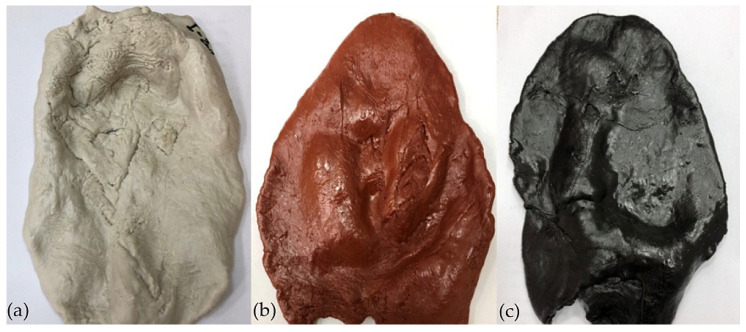
Elastomeric compounds: (**a**) CR/BR/ZnO, (**b**) CR/BR/Fe_2_O_3_, (**c**) CR/BR/CuO.

The first step was the preparation of the pre-mix of the two rubbers on a laboratory two-roll mill with diameters of 200 mm and a length of 400 mm; the rolling temperature was 30–55 °C, and the total time for their preparation was equal to 6 min. The pre-mix thus obtained was divided into three equal parts, each of which was then rolled again to add further ingredients. Stearic acid was added first, followed by the cross-linking agent. The time taken to prepare the mixes was about 4 min. The blends thus obtained were stored separately in foil, at room temperature, without access to light. Vulcanization of the elastomeric blends was carried out on an electrically heated hydraulic press. The compounds were placed between the bays of the press and then cross-linked under a pressure of 180–200 bar. The curing time of all mixes at the four temperatures is summarized in Table 8. The resulting vulcanizates were left to cool for about 10 min and then placed in foil at room temperature for 24 h. Figure 13 presents the tested elastomeric blends.

### 3.3. Evaluation of Vulcanization Process of CR/BR Blends

The curing kinetics of CR/BR composites were determined using an Alpha Technologies (MDR 2000) oscillating disc rheometer (Alpha Technologies, Hudson, OH, USA) at 160 °C (ASTM standard D5289-17 [54]), which was used to determine the following parameters: scorch time (t_02_); vulcanization time (t_90_); minimum torque (T_min_); maximal torque (T_max_); torque increment after 2, 10, and 20 min (ΔT_2,_ ΔT_5,_ ΔT_20_); and cure rate index (CRI). Equations (1) and (2) show the formula for torque increment and cure rate index (CRI):

(1) $$∆T_{n}=T_{n}-T_{min}$$

where n—the vulcanization time in minutes, ΔT—torque increment (dNm), T_n_—torque increment after n minutes (dNm), and T_min_—minimum torque (dNm).

(2) $$CRI=\frac{100}{t_{90}-t_{02}}$$

where CRI—cure rate index, t_02_—scorch time (min), and t_90_—cure time (min).

Based on the study of vulcanization kinetics at four different temperatures (140 °C, 160 °C, 180 °C, and 200 °C), the activation energy for both the vulcanization and scorch time reactions was determined using the Arrhenius equation. This equation describes the relationship between the rate of a reaction and its temperature, assuming the reaction follows first-order kinetics. Equation (3) presents Arrhenius’s formula:

(3) $$\ln k=\ln A-\frac{E}{R\times T}$$

where k—reaction rate constant (s^−1^), A—pre-exponential factor (−), E—activation energy of reaction (J/mol), R—gas constant (J/(mol·K)), and T—temperature (K).

### 3.4. Swelling Properties of CR/BR Vulcanizates

Swelling behavior was assessed using toluene and n-hexane (according to ASTM D471 [55]). From each vulcanizate, four test pieces of 25–60 mg of different shapes were cut out, weighed using an analytical balance, and then swollen in toluene or n-hexane until equilibrium was reached (for 72 h). After this time, the swollen samples were removed from toluene or n-hexane and washed with diethyl ether, and their weights were determined again. The samples were dried to a constant weight at 50 °C and then reweighed.

Equilibrium volume swelling (Q_v_) was calculated using Equation (4):

(4) $$Q_{v}=Q_{w}\times \frac{d_{v}}{d_{s}}$$

where Q_w_—equilibrium mass swelling (mg/mg), d_v_—vulcanizate density (g/mL), and d_s_—solvent density (g/mL).

Equilibrium weight swelling was calculated from Equation (5):

(5) $$Q_{w}=\frac{m_{s}-m_{d}}{m_{d}^{*}}$$

where m_s_—swollen sample weight (mg), m_d_—dry sample weight (mg), and $m_{d}^{*}$—reduced sample weight (mg).

The reduced sample weight was calculated from Equation (6):

(6) $$m_{d}^{*}=m_{d}-m_{0}\times \frac{m_{f}}{m_{t}}$$

where m_0_—initial sample weight (mg), m_f_—filler and inorganic part weight in the sample (mg), and m_t_—total sample weight (mg).

The content of the eluted fraction in solvent (−Q_w_), interpreted as the amount of leaching substances, was calculated from Equation (7):

(7) $$-Q_{w}=\frac{m_{0}-m_{d}^{*}}{m_{0}}$$

The rubber volume fraction (V_R_) was calculated from Equation (8):

(8) $$V_{R}=\frac{1}{1+Q_{v}}$$

The degree of cross-linking (α_c_) was determined using Equation (9):

(9) $$\propto_{c}=\frac{1}{Q_{v}}$$

### 3.5. Mechanical Properties Characterization of CR/BR Vulcanizates

Tensile strength was evaluated using a testing machine (Zwick1435/Roell GmbH & Co. KG, Ulm, Germany). Parameters such as stress at elongation of 100%, 200%, and 300% (Se_100_, Se_200_, Se_300_); tensile strength (TS_b_); and relative elongation at break (E_b_) were determined from that test [56]. Five samples of each vulcanizate were measured, and the test was conducted at a constant speed of 500 mm/min.

The tear strength (T_s_) was assessed according to method A outlined in ISO 34-1:2015 [57], utilizing the same testing machine used for tensile strength determination. Rectangular specimens, sized at 100 mm × 15 mm with a 40 mm cut, were employed for the tests.

The hardness (HA) was measured with a ZwickRoell (Ulm, Germany) hardness tester at ISO 48-4:2018 standard [58]. The samples for this test were prepared in the shape of cylinders in a specially prepared form. The measurement results were determined on the Shore A scale.

### 3.6. Resistance to Thermo-Oxidative Aging of CR/BR Vulcanizates

The vulcanizates contain CR and BR and underwent thermo-oxidating aging in a forced circulating aging oven maintained at 70 °C for 7 days. Following a conditioning period of 24 h at room temperature, alterations in mechanical properties (including stress at 100%, 200%, and 300% strain; tensile strength; and elongation at break) were assessed using the aging factor (AF) according to Formula (10):

(10) $$AF=\frac{{TS}_{b}^{\prime }\times E_{b}^{\prime }}{{TS}_{b}\times E_{b}}$$

where ${TS}_{b}^{\prime }$—tensile strength after thermo-oxidative aging (MPa), TS_b_—tensile strength before thermo-oxidative aging (MPa), $E_{b}^{\prime }$—elongation at break after thermo-oxidative aging (%), and E_b_—elongation at break before thermo-oxidative aging (%).

### 3.7. Assessment of Surface Morphology of CR/BR Vulcanizates

The examination of the vulcanizate morphology was conducted utilizing an inverted scanning electron microscope (SEM), namely, a Hitachi Tabletop Microscope TM-1000 originating from Tokyo, Japan. Sample preparation involved joining the testing sample onto a special table using double-sided self-adhesive foil. A layer of gold was then deposited onto the sample using the Cressington Sputter Coater 108 auto vacuum sputtering machine in Redding, CA, USA, under pressure exceeding 40 mbar for 60 s. Next, the prepared samples were incorporated into the scanning electron microscope chamber for analysis.

### 3.8. Characterization of Chemical Structure with Fourier Transform Infrared Spectroscopy

The infrared spectra of the CR/BR blends were developed with a Thermo Scientific Nicolet 6700 FT-IR spectrometer equipped with a Smart Orbit ATR (Waltham, MA, USA) diamond attachment, using the attenuated total reflectance (ATR) method. The spectra were assessed for the wavenumber range of 4000–400 cm^−1^. Before the spectra of the samples were collected, background measurements were carried out, each time including 64 scans. Identification of the absorbance bandwidth intensities helped compare the characteristic functional groups present in the structures of the tested CR/BR mixes and their vulcanizates.

### 3.9. Evaluation of the Mooney-Rivlin Elasticity Constants of CR/BR Vulcanizates

To determine the elasticity constants 2C_1_ and 2C_2_ of the tested vulcanizates, measuring dumbbells with a width of 6 mm were used. Before starting the test, their thickness was measured to the nearest 0.01 mm, and the length of the testing section, equal to 20 mm, was measured. The specimens were fixed in grips, and the length of the gauge section was read every 30 min with an accuracy of 0.01 mm using a cathetometer, increasing the load on the specimen each time so that the increase in elongation was 15–20%. Elasticity constants were determined using the Mooney–Rivlin Equation (11):

(11) $${2C}_{1}+\lambda^{-1}\times 2C_{2}=\frac{P}{A_{0}\times (\lambda -\lambda^{-2})}$$

where P—deformation force at λ (kG), λ—deformation (λ = l/l_0_), l—length of the loaded sample (cm), l_0_—length of the unloaded sample (cm), A_0_—cross-sectional area of the unloaded sample (cm^2^), 2C_1_—first elastic constant (kG/cm^2^), and 2C_2_—second elastic constant (kG/cm^2^).

### 3.10. Thermal Properties Evaluation of CR/BR Blends

Thermal analysis—thermogravimetric analysis (TGA) and differential scanning calorimetry (DSC)—was performed using a Mettler Toledo TGA/DSC 1 device (Mettler-Toledo, Columbus, OH, USA). TGA analyses were performed using a two-step procedure. First, samples of vulcanizates were heated in the temperature range of 25–600 °C in an argon atmosphere (flow rate 50 mL/min), with a heating rate of 20 °C/min. Next, the gas was pumped into the air (flow rate 50 mL/min), and the heating was continued up to 800 °C, with the same heating rate. DSC measurements were performed on rubber blends. Samples were heated from −100 °C to 250 °C, with a heating rate of 10 °C/min. Liquid nitrogen was applied to cool the sample before the measurement.

## 4. Conclusions

The use of ZnO, Fe_2_O_3_, or CuO made it possible to cross-link blends of chloroprene rubber and butadiene rubber at different temperatures. The study of cross-linking kinetics showed that, regardless of the type of metal oxide used to cross-link the CR/BR compound, as the curing temperature increased, the values of scorch time and curing time decreased. Reducing the vulcanization time reduced the cost of manufacturing rubber products, but a lower scorch time adversely affected process safety. Based on the results of the activation energy of scorch and vulcanization, it was found that the activity of ZnO and Fe_2_O_3_ was higher than that of CuO. For the various oxides, the torque increment varied depending on the curing temperature. For iron(III) oxide cross-linked vulcanizates, higher curing temperatures resulted in a decrease in torque increment, while the opposite trend was observed for CR/BR/CuO and CR/BR/ZnO. As the vulcanization temperature increased, the degree of cross-linking of CR/BR/ZnO and CR/BR/CuO compositions increased. The opposite results were observed for CR/BR/Fe_2_O_3_. The calculated elasticity constants showed that, despite a lower degree of cross-linking than for compositions with iron(III) oxide and copper(II) oxide, those cross-linked with zinc oxide formed a more proper structure. CR/BR vulcanizates cross-linked with Fe_2_O_3_ exhibited the highest tensile strength before thermo-oxidative aging, but both CR/BR/Fe_2_O_3_ and CR/BR/CuO compositions showed poorer aging resistance than CR/BR/ZnO due to their multiple oxidation states. At higher curing temperatures, the sample cross-linked with ZnO achieved the greatest tensile and tear strength, while specimens cross-linked with CuO showed the opposite trend. The highest hardness was recorded for CR/BR/CuO-140, although increasing the curing temperature led to decreased hardness, an unusual behavior for elastomers. CR/BR/ZnO and CR/BR/Fe_2_O_3_ vulcanizates showed maximum hardness at a curing temperature of 160 °C.

DSC analysis revealed similar glass transition temperatures (~36 °C) across all samples, indicating good miscibility and uniform blending. CR/BR/Fe_2_O_3_ demonstrated the most efficient cross-linking with the highest enthalpy release, while CR/BR/CuO was the least effective. TGA showed that CR/BR/ZnO samples had the greatest mass loss (~50%), though all samples exhibited similar degradation above 500 °C. FTIR confirmed cross-linking via characteristic absorbance changes, with similar network structures observed across all samples. SEM analysis showed no visible phase separation, supporting good compatibility and the formation of interelastomeric bonds, consistent with the single T_g_ observed in DSC. In summary, the composition of chloroprene rubber and butadiene rubber was cross-linked with zinc, iron(III), or copper(II) oxides. The type of oxide and the vulcanization temperature affected the curing efficiency and properties of the vulcanizates. Considering the search for more sustainable alternatives to zinc oxide in the cross-linking of elastomeric blends, iron(III) oxide was worth noting, as it allowed efficient curing at lower temperatures and resulted in improved mechanical properties and an optimal degree of cross-linking.

## Figures and Tables

**Table 1:** Results of vulcametric parameters of CR/BR blends.

Curing Agent	T_v_ (°C)	T_min_ (dNm)	ΔT_2_ (dNm)	ΔT_10_ (dNm)	ΔT_20_ (dNm)	t_02_ (min)	t_90_ (min)	CRI (min^−1^)
ZnO	140	0.61	1.24	2.79	2.86	1.5	19.6	5.5
	160	0.58	2.38	2.66	2.99	1.0	17.6	6.0
	180	0.55	2.19	2.76	3.07	0.5	11.3	9.2
	200	0.59	2.23	3.07	3.3	0.2	9.5	10.8
Fe_2_O_3_	140	0.56	0.61	5.17	5.39	5.9	8.7	35.7
	160	0.48	3.86	5.13	5.1	1.8	2.6	125.0
	180	0.39	5.07	4.91	4.98	0.8	1.1	333.3
	200	0.38	4.88	4.92	4.88	0.5	0.7	500.0
CuO	140	0.58	0.79	1.51	2.09	4.0	36.9	3.0
	160	0.52	1.08	2.56	3.72	2.0	21.8	5.1
	180	0.48	1.75	4.06	4.53	1.0	12.5	8.7
	200	0.45	2.86	4.42	4.81	0.6	9.0	11.9

T_v_—temperature of vulcanization, T_min_—minimal torque, ΔT_n_—torque increment after n minutes, t_02_—scorch time, t_90_—cure time, CRI—cure rate index.

**Table 2:** Activation energy values of scorch and vulcanization.

Symbol	E_scr._ (kJ/mol)	E_vulc._ (kJ/mol)
CR/BR/ZnO	54.28	21.23
CR/BR/Fe_2_O_3_	67.07	69.98
CR/BR/CuO	81.91	38.97

E_scr._—scorch activation energy, E_vulc._—vulcanization activation energy.

**Table 3:** Results of the swelling properties of the CR/BR vulcanizates and their degree of cross-linking.

Curing Agent	T_v_ (^°^C)	Toluene				n-Hexane		
		Q_v_^T^ (mL/mL)	−Q_w_^T^ (mg/mg)	V_R_^T^ (−)	α_c_ (−)	Q_v_^H^ (mL/mL)	−Q_w_^H^ (mg/mg)	V_r_^H^ (−)
ZnO	140	8.06 ± 0.18	0.23 ± 0.01	0.110 ± 0.002	0.124 ± 0.03	1.24 ± 0.02	0.07 ± 0.01	0.447 ± 0.004
	160	8.24 ± 0.13	0.22 ± 0.01	0.108 ± 0.002	0.121 ± 0.002	1.28 ± 0.02	0.08 ± 0.01	0.438 ± 0.004
	180	7.61 ± 0.10	0.19 ± 0.01	0.116 ± 0.001	0.131 ± 0.002	1.21 ± 0.02	0.07 ± 0.01	0.453 ± 0.005
	200	7.26 ± 0.29	0.15 ± 0.01	0.121 ± 0.004	0.138 ± 0.005	1.18 ± 0.03	0.06 ± 0.01	0.459 ± 0.006
Fe_2_O_3_	140	5.63 ± 0.10	0.06 ± 0.01	0.151 ± 0.002	0.178 ± 0.003	0.97 ± 0.04	0.06 ± 0.01	0.508 ± 0.010
	160	5.70 ± 0.02	0.06 ± 0.01	0.149 ± 0.001	0.176 ± 0.001	1.00 ± 0.03	0.06 ± 0.01	0.500 ± 0.008
	180	5.71 ± 0.06	0.06 ± 0.01	0.149 ± 0.001	0.175 ± 0.002	1.01 ± 0.02	0.05 ± 0.01	0.500 ± 0.006
	200	6.26 ± 0.06	0.07 ± 0.01	0.138 ± 0.001	0.160 ± 0.002	1.00 ± 0.03	0.05 ± 0.01	0.500 ± 0.008
CuO	140	8.65 ± 0.03	0.25 ± 0.01	0.111 ± 0.003	0.116 ± 0.001	1.21 ± 0.02	0.06 ± 0.01	0.452 ± 0.004
	160	7.35 ± 0.13	0.12 ± 0.01	0.120 ± 0.002	0.136 ± 0.002	1.02 ± 0.01	0.05 ± 0.01	0.495 ± 0.002
	180	6.25 ± 0.03	0.07 ± 0.01	0.138 ± 0.001	0.160 ± 0.001	0.83 ± 0.01	0.03 ± 0.01	0.546 ± 0.004
	200	5.14 ± 0.09	0.05 ± 0.01	0.163 ± 0.002	0.195 ± 0.003	0.77 ± 0.04	0.05 ± 0.01	0.566 ± 0.013

T_v_—temperature of vulcanization; Q_V_^T^, Q_V_^H^—equilibrium volume swelling, in toluene and n-hexane, respectively; −Q_W_^T^, −Q_W_^H^—amount of leached fraction in toluene and n-hexane, respectively; V_R_^T^, V_R_^H^—volume proportion of rubber in swollen sample, in toluene and n-hexane, respectively; α_c_—degree of cross-linking.

**Table 4:** Constant elasticities of CR/BR vulcanizates.

Curing Agent	T_v_ (°C)	2C_1_ (kG/cm^2^)	2C_2_ (kG/cm^2^)
ZnO	140	2.67 ± 0.40	1.65 ± 0.25
	160	3.07 ± 0.46	1.76 ± 0.26
	180	3.67 ± 0.55	0.86 ± 0.13
	200	3.75 ± 0.56	0.87 ± 0.13
Fe_2_O_3_	140	3.93 ± 0.59	1.61 ± 0.24
	160	3.61 ± 0.54	2.32 ± 0.35
	180	3.21 ± 0.48	2.31 ± 0.35
	200	4.03 ± 0.60	2.63 ± 0.39
CuO	140	2.33 ± 0.35	3.55 ± 0.53
	160	3.61 ± 0.54	4.54 ± 0.68
	180	3.99 ± 0.60	2.51 ± 0.38
	200	4.25 ± 0.64	0.94 ± 0.14

T_v_—temperature of vulcanization; 2C_1_, 2C_2_—first and second Mooney–Rivlin elasticity constants, respectively.

**Table 5:** Results of tensile strength properties of CR/BR vulcanizates before and after thermo-oxidative aging.

Curing Agent	T_v_ (^°^C)	S_E100_ (MPa)	S′_E100_ (MPa)	TS_b_ (MPa)	TS′_b_ (MPa)	E_b_ (%)	E′_b_ (%)	AF (−)
ZnO	140	0.56 ± 0.06	0.75 ± 0.01	6.93 ± 0.25	7.56 ± 0.20	1014 ± 470	739 ± 18	0.79
	160	0.79 ± 0.11	0.77 ± 0.04	4.89 ± 0.45	6.25 ± 1.13	838 ± 108	699 ± 41	1.07
	180	0.64 ± 0.04	0.78 ± 0.01	6.85 ± 0.89	6.43 ± 0.31	1080 ± 42	687 ± 9	0.60
	200	0.58 ± 0.01	0.72 ± 0.02	7.29 ± 0.82	5.56 ± 0.41	1199 ± 67	657 ± 16	0.42
Fe_2_O_3_	140	0.70 ± 0.03	1.14 ± 0.02	11.40 ± 0.20	1.20 ± 0.06	1268 ± 14	103 ± 15	0.01
	160	0.69 ± 0.03	1.15 ± 0.04	8.86 ± 0.39	1.26 ± 0.07	1159 ± 10	118 ± 9	0.01
	180	0.63 ± 0.01	1.09 ± 0.01	10.30 ± 0.10	1.27 ± 0.04	1192 ± 37	133 ± 6	0.01
	200	0.63 ± 0.03	1.05 ± 0.01	9.17 ± 1.01	1.27 ± 0.04	1182 ± 74	146 ± 11	0.02
CuO	140	0.67 ± 0.11	0.53 ± 0.03	5.45 ± 0.25	1.44 ± 0.12	1287 ± 5	348 ± 28	0.07
	160	0.60 ± 0.02	0.62 ± 0.01	8.09 ± 0.30	1.42 ± 0.06	1288 ± 1	306 ± 13	0.04
	180	0.60 ± 0.04	0.67 ± 0.01	9.53 ± 1.08	1.60 ± 0.11	1226 ± 88	303 ± 15	0.04
	200	0.94 ± 0.09	0.76 ± 0.01	2.59 ± 0.15	1.74 ± 0.16	393 ± 8	267 ± 19	0.46

T_v_—temperature of vulcanization; S_E100_—stress at an elongation of 100%; TS_b_—tensile strength; E_b_—elongation at break; S′_E100_—stress at an elongation of 100% after thermo-oxidative aging; TS′_b_—tensile strength after thermo-oxidative aging; E′_b_—elongation at break after thermo-oxidative aging; AF—aging factor.

**Table 6:** Temperature and enthalpy of cross-linking determined by DSC for the CR/BR blends cross-linked at 180 °C.

Symbol	T_g_ (°C)	T_c_ (°C)	T_V_ range (°C)	ΔH (J/g)
CR/BR/ZnO	−36.2	142.5	118–159	6.34
CR/BR/Fe_2_O_3_	−36.5	168.8	154–178	13.43
CR/BR/CuO	−35.,4	125.1	109–138	1.09

T_g_—glass transition temperature, T_c_—optimal cross-linking temperature, T_V_ range—cross-linking temperature range, ΔH—enthalpy of cross-linking.

**Table 7:** Formulations of CR/BR compounds.

Ingredient	Weight Parts (phr)		
CR	75	75	75
BR	25	25	25
ZnO	3	----	----
Fe_2_O_3_	----	3	----
CuO	----	----	3
SA	1	1	1
Determination	CR/BR/ZnO	CR/BR/Fe_2_O_3_	CR/BR/CuO

CR—chloroprene rubber, BR—butadiene rubber, ZnO—zinc oxide, Fe_2_O_3_—iron(III) oxide, CuO—copper(II) oxide, SA—stearic acid.

**Table 8:** Curing time of CR/BR blends and their determinations.

Determination	Curing Temperature (°C)	Curing Time (min)
CR/BR/ZnO	140	10
	160	10
	180	10
	200	10
CR/BR/Fe_2_O_3_	140	10
	160	2
	180	2
	200	2
CR/BR/CuO	140	20
	160	20
	180	20
	200	20

## Data Availability

The original contributions presented in the study are included in the article, and further inquiries can be directed to the corresponding author.

## Abbreviations

The following abbreviations are used in this manuscript:

NR	Natural rubber
SBR	Styrene-butadiene rubber
CR	Chloroprene rubber
EPDM	Ethylene-propylene-diene rubber
MVQ	Methyl vinyl silicon
FKM	Fluorocarbon rubber
BR	Butadiene rubber
SBS	Styrene-butadiene-styrene
PCP	Phosphorylated cardanol prepolymer
EPM	Ethylene-propylene rubber
HNBR	Hydrogenated nitrile-butadiene rubber
CSM	Chlorosulfonated polyethylene
XNBR	Carboxylated nitrile-butadiene rubber
XSBR	Carboxylated styrene–butadiene rubber
CR/BR/ZnO	Chloroprene/butadiene rubber blend cross-linked with zinc oxide
CR/BR/Fe_2_O_3_	Chloroprene/butadiene rubber blend cross-linked with iron(III) oxide
CR/BR/CuO	Chloroprene/butadiene rubber blend cross-linked with copper(II) oxide
CR/BR/ZnO-140	Chloroprene/butadiene rubber vulcanizate cross-linked with zinc oxide at 140 °C
CR/BR/ZnO-160	Chloroprene/butadiene rubber vulcanizate cross-linked with zinc oxide at 160 °C
CR/BR/ZnO-180	Chloroprene/butadiene rubber vulcanizate cross-linked with zinc oxide at 180 °C
CR/BR/ZnO-200	Chloroprene/butadiene rubber vulcanizate cross-linked with zinc oxide at 200 °C
CR/BR/Fe_2_O_3_-140	Chloroprene/butadiene rubber vulcanizate cross-linked with iron(III) oxide at 140 °C
CR/BR/Fe_2_O_3_-160	Chloroprene/butadiene rubber vulcanizate cross-linked with iron(III) oxide at 160 °C
CR/BR/Fe_2_O_3_-180	Chloroprene/butadiene rubber vulcanizate cross-linked with iron(III) oxide at 180 °C
CR/BR/Fe_2_O_3_-200	Chloroprene/butadiene rubber vulcanizate cross-linked with iron(III) oxide at 200 °C
CR/BR/CuO-140	Chloroprene/butadiene rubber vulcanizate cross-linked with copper(II) oxide at 140 °C
CR/BR/CuO-160	Chloroprene/butadiene rubber vulcanizate cross-linked with copper(II) oxide at 160 °C
CR/BR/CuO-180	Chloroprene/butadiene rubber vulcanizate cross-linked with copper(II) oxide at 180 °C
CR/BR/CuO-200	Chloroprene/butadiene rubber vulcanizate cross-linked with copper(II) oxide at 200 °C
ZnO	Zinc oxide
MgO	Magnesium oxide
Fe_2_O_3_	Iron(III) oxide
Fe_3_O_4_	Iron(II,III) oxide
CuO	Copper(II) oxide
Ag_2_O	Silver(I) oxide
nZnO	Nano zinc oxide
SA	Stearic acid
t_02_	Scorch time
t_90_	Cure time
ΔT_n_	Torque increment after n minutes
T_v_	Temperature of vulcanization
T_min_	Minimal torque
T_max_	Maximal torque
CRI	Cure rate index
E_scr._	Scorch activation energy
E_vulc._	Vulcanization activation energy
k	Reaction rate constant
A	Pre-exponential factor
E	Activation energy of the reaction
R	Gas constant
T	Temperature
Q_V_^T^, Q_V_^H^	Equilibrium volume swelling in toluene or n-hexane, respectively
Q_w_	Equilibrium mass swelling
d_v_	Density of vulcanizate
d_s_	Density of solvent
m_s_	Swollen sample weight
m_d_	Dry sample weight
m^*^_d_	Reduced sample weight
m_0_	Initial sample weight
m_f_	Filler and inorganic part weight in the sample
m_t_	Total sample weight
−Q_w_^T^, −Q_w_^H^	Amount of leached fraction in toluene and n-hexane, respectively
V_R_^T^, V_R_^H^	Volume proportion of rubber in swollen sample, in toluene and n-hexane, respectively
α_c_	Degree of cross-linking
2C_1_	First elasticity constant
2C_2_	Second elasticity constant
P	Deformation force
λ	Deformation
A_0_	Cross-sectional area of the unloaded sample
l	Length of the loaded sample
l_0_	Length of the unloaded sample
S_E100_	Stress at elongation 100% before aging
S′_E100_	Stress at elongation 100% after aging
TS_b_	Tensile strength before aging
${TS}_{b}^{\prime }$	Tensile strength after aging
E_b_	Elongation at break before aging
$E_{b}^{\prime }$	Elongation at break after aging
AF	Aging factor
HA	Hardness in the Shore A scale
T_s_	Tear strength
DSC	Differential scanning calorimetry
TGA	Thermogravimetric analysis
T_g_	Glass transition
T_c_	Optimal cross-linking temperature
T_v_ range	Vulcanization temperature range
ΔH	Enthalpy of cross-linking
DTG	Differential thermogravimetric analysis
FT-IR	Fourier transform infrared spectroscopy
SEM	Scanning electron microscopy
